# Factors facilitating or hindering meaningful staff–client interactions in people with intellectual disabilities and challenging behaviour: A systematic mixed studies review using thematic synthesis

**DOI:** 10.1111/jar.12830

**Published:** 2020-11-20

**Authors:** M.A.G. Simons, R. Koordeman, A.P.A.M. Willems, M. Hermsen, L.M. Rooijackers, R. Otten

**Affiliations:** ^1^ Research and Development Pluryn Nijmegen The Netherlands; ^2^ Behavioural Science Institute Radboud University Nijmegen The Netherlands; ^3^ Centre of Expertise Koraal Heel the Netherlands; ^4^ Research Centre for Social Support and Community Care Nijmegen The Netherlands; ^5^ REACH Institute Arizona State University Tempe AZ USA

**Keywords:** challenging behaviour, client interaction, intellectual disability, professional caregivers, staff

## Abstract

**Background:**

Interactions with professional caregivers affect the quality of support and life of people with intellectual disabilities and contribute to the occurrence of challenging behaviour. The present literature review provides an overview of factors facilitating or hindering meaningful staff–client interactions in people with borderline to profound intellectual disabilities and challenging behaviour.

**Method:**

Database searches, reference list and citation screening, and expert consultations were undertaken. A thematic synthesis of 28 studies was performed.

**Results:**

Factors were identified at the client (i.e. behaviour, emotions and (dis)abilities), staff (i.e. interactive principles, knowledge, psychological resources, attributions, attitudes and (coping with) emotions) and context levels (i.e. group size, team and organization).

**Conclusions:**

The present overview provides insights into factors that facilitate or hinder meaningful staff–client interactions with people with intellectual disabilities and challenging behaviour. The results support the need to combine client, staff and contextual factors when considering staff–client interactions in research and practice.

## 1 INTRODUCTION

People with intellectual disabilities often depend on the support of professional caregivers due to deficits in cognitive, social and adaptive functioning (Schalock et al., 2010). In the field of professional caregiving to people with intellectual disabilities, the importance of clients’ quality of life (QOL) is increasingly recognized, with interpersonal relations as the most frequently cited indicator of clients’ QOL (Schalock, 2004). Considering the importance of interpersonal relations to the well‐being of clients, the field of care ethics calls for interpersonal staff–client relations as an essential condition for quality care rather than standardization and producing measurable outcomes (i.e. professional loving care approach: Hermsen et al., 2014). These relationships result from repeated meaningful staff–client interactions. ‘Meaningful’ interactions are functional, pleasing and important to the client (Carr et al., 2016) and enable staff to gain insight in, and respond to, the needs of the clients (Reinders, 2010). As interpersonal relationships are essential to the quality of care and clients’ QOL, factors that affect meaningful staff–client interactions are important to study.

One of the challenges staff face in interaction with clients with intellectual disabilities is that in residential settings approximately 20% of the clients shows severe and enduring challenging behaviour, most commonly aggression or self‐injury (Healthcare Inspectorate, 2005). Importantly, what is understood as ‘challenging’ depends on cultural norm and the social context is key to understanding why challenging behaviour occurs and is maintained. A person does not ‘have’ challenging behaviour, but a person displays behaviour that is experienced as challenging by others in certain circumstances (Burton, 2001; Hastings et al., 2013). Following an extension of the model of human functioning of people with an intellectual disability as introduced by the American Association on Intellectual and Development Disabilities (i.e. the AAIDD‐model; Schalock et al., 2010), challenging behaviour can be understood as a signal of an imbalance between support from caregivers and the person's (dis)abilities within five dimensions of human functioning (i.e. cognitive functioning, adaptive behaviour, participation, health, and the physical and social context) while meaningful interactions reflect a balance between clients’ needs and caregiver support (Embregts et al., 2019). It is generally acknowledged that the behaviour of these caregivers affects the origin and maintenance of challenging behaviour (Hastings et al., 2013; Hastings & Remington, 1994). The occurrence of challenging behaviour is on the one hand affected by staffs’ immediate responses to incidents of challenging behaviour (Hastings et al., 2013; Hastings & Remington, 1994) and, on the other hand, by the day‐to‐day interactions with clients outside the immediate occurrence of challenging behaviour (Hastings & Remington, 1994). Previous research has mainly focused on staffs’ immediate responses to incidents of challenging behaviour, including (a) (correlates of) staff responses (e.g. attitudes, emotions), (b) trainings to modify staff responses and (c) interventions in response to challenging behaviour (Allen, 2001; Cox et al., 2015; Hastings, 2005; Hastings & Remington, 1994). The present review addresses factors that affect meaningful interactions between staff and clients.

Unfortunately, shortcomings in staff–client interactions have been reported, related to infrequency, short durations and a lack of responsivity to clients’ needs (Hastings & Remington, 1994; McConkey et al., 1999), and the residential settings most clients with intellectual disabilities and challenging behaviour reside in present challenges to engage in meaningful interactions due to staffs’ high workload and frequent changes (Schuengel et al., 2010). The inadequate interactions contribute to challenging behaviour in several possible ways: (a) the absence of interaction results in challenging behaviour to self‐stimulate or to obtain staff contact, (b) challenging behaviour serves a communicative function when clients lack more effective communication, resulting from a mismatch between staff receptive and expressive communication and clients’ abilities and needs and (c) challenging behaviour is a way to cope with stress in face of insufficient coping skills and a lack of interpersonal relationships to rely on in stressful situations (Hastings & Remington, 1994; Kevan, 2003; Schuengel et al., 2010). Increases in the rate of staff–client interactions have been shown to potentially reduce challenging behaviour, depending on the function challenging behaviour serves (e.g. attain versus avoid social contact). Rather than merely increasing the rate of interactions, it is important to promote meaningful interactions that fit the clients’ needs (Hastings & Remington, 1994).

Day‐to‐day interactions between staff and clients are not per se meaningful (Reinders, 2010). Whether staff is able to engage in meaningful interactions with clients with intellectual disabilities and challenging behaviour depends on staffs’ extraordinary sensitivity and interest in a client (Reinders, 2010; Schuengel et al., 2010). This sensitivity and interest is paramount as challenging behaviour co‐occurs with impairments in communicative abilities. The subtle cues clients use to communicate are difficult to interpret and staffs’ messages are at risk of being misunderstood, easily resulting in a mutual misunderstanding (Kevan, 2003). Meaningful interactions based on sensitivity and interest result in a mutual understanding that enables staff to attune their support efforts to clients’ needs. In this sense, staffs’ intentionality of being attached and attuned to a particular client is key to engaging in meaningful interactions, which results from an interplay between individual factors (i.e. the quality of relationships with a client varies among staff members) and context factors (i.e. organizations facilitate or hinder staffs’ intentionality) (Schuengel et al., 2010).

While the importance of meaningful interactions between staff and clients is recognized, inadequate interactions have been reported and insights in the factors that affect meaningful interactions remain scarce. The present review aimed to thematically synthesize the qualitative and quantitative literature on factors that facilitate or hinder meaningful interactions between staff and clients with borderline to profound intellectual disabilities and challenging behaviour in residential settings.

## 2 METHODS

An electronic literature search was undertaken using the databases Web of Science, ERIC and PsycINFO in February 2019. The search covered synonyms for the following key topics: (a) intellectual disability, (b) challenging behaviour and (c) staff–client interactions (Table 1 presents an overview of search terms). Titles and abstracts were screened for relevance by one author (MS). Full texts were screened for relevance by two authors independently (RK, MS). Discrepancies were resolved by discussion. A second literature search was performed in July 2020 by the first author, adding the search terms ‘staff contact’, ‘staff attention’ and ‘rapport’ to the third key topic. Reference lists and citations of included articles were searched, and experts in the field were consulted.

**Table 1:** Search terms

Key topic 1: Intellectual disability	Key topic 2: Challenging behaviour	Key topic 3: Staff–client interaction
Intellectual disability	Challenging behaviour	Interaction
Developmental disability	Maladaptive behaviour	Dyad
Intellectual impairment	Aberrant behaviour	Communication
Mental retardation	Disruptive behaviour	Relationship
Mental handicap		Staff contact*
Mental deficiency		Staff attention*
Learning disability		Rapport*

Note

Suffix variations were accounted for by the asterisk symbol. The search string was constructed by combining the search terms in each column by “OR” and the key topics in each row by “AND”, as below:

(‘intellectual* disab*’ OR ‘developmental* disab*’ OR ‘intellectual* impair*’ OR ‘mental* retar*’ OR ‘mental* handicap*’ OR ‘ mental* deficien*’ OR ‘learning disab*’) AND (‘challenging behavio*’ OR ‘maladaptive behavio*’ OR ‘aberrant behavio*’ OR ‘disruptive behavio*’) AND (‘interaction*’ OR ‘dyad*’ OR ‘communicati*’ OR ‘relationship*’ OR ‘staff contact*’ OR ‘staff attention*’ OR ‘rapport’). *Search terms added in the second literature search in July 2020.

Studies were included if they met the following criteria: (a) the target group involved clients with intellectual disabilities and challenging behaviour, (b) empirical studies were reported in English, (c) studies yielded information about factors that affect staff–client interactions, (d) studies were conducted in residential settings or day‐care centres and (e) studies were published between January 1990 and July 2020. Exclusion criteria included as follows: (a) studies concerned a target group with motor disabilities, (b) studies concerned interaction with a partner other than staff, (c) non‐empirical studies, (d) studies concerned immediate responses to challenging behaviour without referring to staff–client interactions, (e) studies compared hospital wards with specialized houses in terms of quantity of staff contact without referring to factors that affect differences in staff contact or (f) studies used vignettes or simulations.

### 2.1 Analysis

Data were extracted and analysed using data‐driven thematic synthesis as described by Thomas and Harden (2008) using Atlas ti.8. Included studies did not allow for quantitative analysis due to the variation in outcome measures, small sample sizes and the mix of qualitative and quantitative designs.

First, result and discussion sections of all included studies were coded line‐by‐line, to form codes from segments of text. Codes were created inductively to capture the meaning of segments of text, and no priori coding structure was employed. Codes were structured as ‘free codes’ that is without a hierarchical structure. One or more codes were applied to segments of text that were relevant to the research question. Second, codes were clustered into related areas to develop descriptive themes. New codes were created to cluster initial codes with similar meanings, and a hierarchical structure was formed in which related codes were clustered into themes. Third, analytical themes were generated by interpreting the descriptive themes beyond the content of the original studies and considering the implications of the descriptive themes for the review question. The hierarchical structure of themes and codes was refined.

## 3 RESULTS

**Figure 1:**
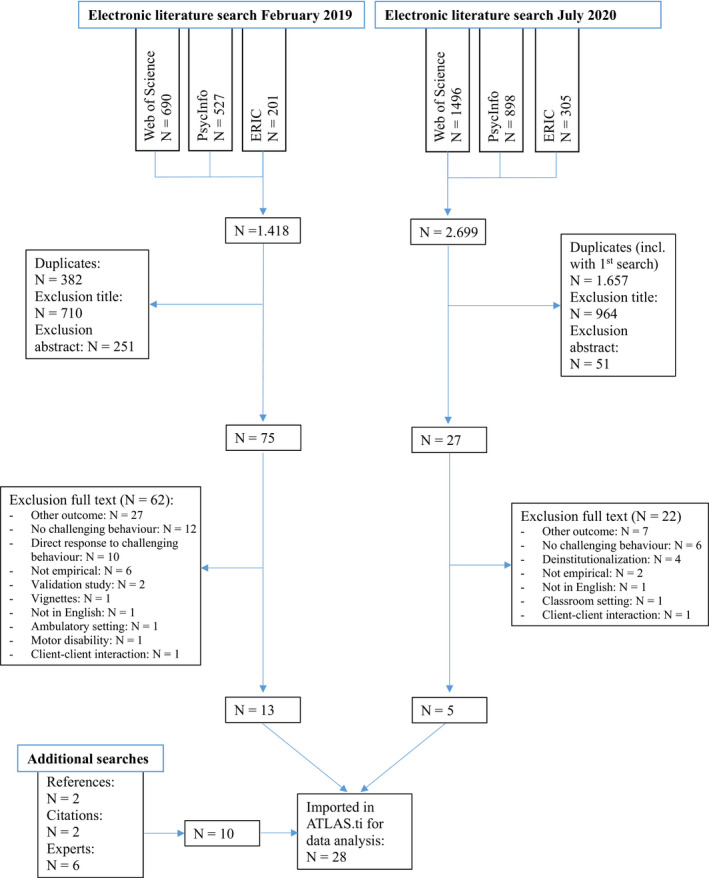
Flow diagram [Colour figure can be viewed at wileyonlinelibrary.com]

The electronic literature search resulted in 1036 unique articles, of which 13 articles met the selection criteria. Inter‐rater agreeability for full‐text screening was 82%. The second literature search identified 1042 unique articles, of which five articles met the selection criteria. Ten articles were identified from additional searches, resulting in 28 included articles (Figure 1).

### 3.1 Study characteristics

The characteristics of included studies are presented in Table 2 (16 quantitative studies) and Table 3 (11 qualitative studies and one mixed‐method study). The studies included the following participants: direct care staff (*N* = 18), clients with intellectual disabilities and challenging behaviour (*N* = 8), staff–client dyads (*N* = 4) and/or other professionals (*N* = 3). Sample sizes ranged from 3 to 318. The studies involved clients with mild to profound intellectual disabilities (*N* = 12), severe or profound intellectual disabilities (*N* = 5), borderline, mild or moderate intellectual disabilities (*N* = 4) or a not reported level of intellectual disabilities (*N* = 7). Methods used included (video)observations (*N* = 13), interviews (*N* = 12), questionnaires (*N* = 9) and document analysis (*N* = 2).

**Table 2:** Characteristics of quantitative studies

1^st^ (, 2^nd^) author, year (place)	Aim/outcome	Participants (*n*)
1. Allen, 1999 (England)	Engagement levels, rates of staff contact and the nature of activities undertaken in a sample of clients receiving day services in two English counties	Adults (20) with borderline‐mild (30%), severe‐profound (50%) or unclassified (20%) intellectual disabilities and high rates of challenging behaviour
2. Embregts, 2019 (Netherlands)	Effects of a training program on staffs’ emotional intelligence and awareness of their behaviour	Dyads (29) of staff and children/adolescents/adults with mild intellectual disbalities
3. Felce, 1991 (England)	Relationships between staff–client interactions, client responding and group sizes	Adults (90) with severe‐profound intellectual disabilities and staff
4. Felce, Perry, 1995 (England)	Availability of assistance/opportunities to engage in activities for clients	Staffed houses (15) for adults (54) with severe‐profound intellectual disabilities
5. Felce, Lowe, 1995 (England)	Client engagement, staff behaviour, and quality and quantity of staff–client interaction	Adults (16) with severe intellectual disabilities
6. Emerson, 1992 (England)	Resident and staff activity in two hospital‐based staffed houses	Two staffed houses serving adults (8) with severe intellectual disabilities
7. McLaughlin, 2005 (VS)	Evaluating the effectiveness of a multicomponent intervention to improve rapport between clients and their caregiver	Staff–client dyads (3) with intellectual disabilities
8. Walz, 1992 (England)	Staff attitudes and staff–client interactions in formal versus informal setting	Staff (17) of adults (9) with mild intellectual disabilities
9. Willems, 2010 (Netherlands)	Relation between intrapersonal and interpersonal staff behaviours, accounting for background variables of clients and staff	Direct care staff and occupational therapy staff (292) of clients with mild‐profound intellectual disabilities
10. Willems, 2014 (Netherlands)	Influence of challenging behaviour, interpersonal attitude and emotional intelligence on staff interactive behaviour	Direct care staff and occupational therapy staff (158) of individuals with mild‐profound intellectual disabilities
11. Willems, 2016 (Netherlands)	Influence of client interpersonal behaviour, staff reactions to challenging behaviour, staff psychological resources and staff contact on staff interpersonal behaviour	Staff (318) of children/adults with mild‐profound intellectual disabilities
12. Willems, 2018 (Netherlands)	Dynamical patterns of staff–client interaction	Staff (3) of a fourteen‐year‐old girl with moderate intellectual disabilities
13. Wolff, 2012 (VS)	Caregiver responses to adaptive client behaviour	Adults with moderate‐profound intellectual disabilities and self‐injurious behaviour (89), control group without self‐injurious behaviour (20) and staff
14. Zijlmans, 2012 (Netherlands)	Relationship between staffs’ experienced emotions, causal attributions, and interpersonal style, and the influence of type of challenging behaviour on these factors	Staff (99) of children/adults with borderline‐profound intellectual disabilities
15. Zijlmans, 2014a (Netherlands)	Relationship between levels of staff engagement and avoidance with challenging and desirable behaviours and clients’ initiatives for contact	Staff (8) and adults (3) with mild‐severe intellectual disabilities
16. Zijlmans, 2014b (Netherlands)	Effectiveness of a training programme on staff–client interactions	Staff–client dyads (37) with mild‐severe intellectual disabilities

**Table 3:** Characteristics of qualitative and mixed‐method studies

1st author, year (place)	Aim/outcome	Participants (*n*)
17. Antonsson, 2008 (Sweden)	Carers’ reflections on their interaction with clients	Staff (16) of adults with moderate (2), severe (6) and profound (3) intellectual disabilities
18. Antonsson, 2013 (Sweden)	Factors constituting skilled interaction	Staff (16) of adults with moderate (2), severe (6) and profound (3) intellectual disabilities
19. Antonsson, 2016 (Sweden)*	Evaluate a web‐based intervention on staff abilities to interact	Staff (7) of an adult (1) with severe intellectual disabilities
20. Bambara, 2001 (VS)	Experiences and perspectives of team members that successfully implemented positive approaches	Core team members (19) of successful teams (4)
21. Bradshaw, 2013 (England)	Staff insights in development of skills over time	Staff (14)
22. Clarke, 2017 (England)	Explore understanding of service‐users around their behaviour, what shaped these understandings, and the relationship between how behaviours are managed and well‐being	Adults (8) with mild‐moderate intellectual disabilities
23. Olivier‐Pijpers, 2020 (Netherlands)	Explore the perspectives of residents and their representatives on the influence of the organizational environment on challenging behaviour	Clients and their representatives (16) with mild‐severe intellectual disabilities
24. Knotter, 2018 (Netherlands)	Factors related to negative and constructive staff–client interactions	Experts of the Centre for Consultation and Expertise (5)
25. Nagra, 2017 (England)	Staff views of the effectiveness of training in intensive interaction	Staff (8) trained in II
26. Ravoux, 2012 (England)	Staff perspective on managing challenging behaviour	Staff (11) of clients with mild‐severe intellectual disabilities
27. Thompson, 2019 (England)	Evaluate whether and how discovery awareness is helpful for staff, and whether it increases their self‐efficacy in supporting people with challenging behaviour	Members (40) of multidisciplinary teams supporting clients with intellectual disabilities and challenging behaviour who took part in a discovery awareness meeting
28. Whittington, 2005 (England)	Staff beliefs about challenging behaviour and development of beliefs over time	Staff (18)

### 3.2 Staff–client interactions

Three levels of facilitating and hindering factors to building meaningful staff–client interactions were identified in the thematic synthesis, namely, client, staff and context level. Table 4 presents an overview of the factors per level.

**Table 4:** Overview of facilitating and hindering factors on client, staff and context level

(Sub)categories	Facilitating	Hindering
Client		
Behaviour	Social/communicative behaviour (5, 15, 16) Internalizing challenging behaviour (11) Rate of severe challenging behaviour (6)	General occurrence challenging behaviour (4, 6, 9, 10, 15) Severity self‐injurious behaviour (14) Externalizing challenging behaviour (13)
Emotions	Safety, security, confidence (17, 18, 20, 25) Able to express emotions (18, 25) Support to regulate emotions (22)	
(Dis)abilities	Severity of the intellectual disability (11)	Severity of the intellectual disability(4, 15)
Staff		
Interactive principles	Autonomy with boundaries (2, 16, 17, 18, 20, 21, 28) Relatedness (2, 16, 17, 21, 25) Competence (2, 16, 17, 18,25) Confirmation (18, 20, 21, 24) Humour (17, 25) Trust (20, 22, 23, 26) Proximity (17, 20) Synchronization (11, 12) Reciprocity (7, 18, 23)	Doing things for a client he/she can do him/herself (21) Neglecting individuality (21)
Knowledge	Spending time together (18, 20, 21) Life history and experiences (17, 18, 24) Talking to/observing others (21, 26, 28)	Lack of knowledge about treatment programme (24)
Psychological resources	Enthusiasm (24) Patience (18, 24, 25) Flexibility (17, 18, 20, 24) Confidence (19, 25, 26, 27) Self‐efficacy (11) Self‐reflection (11, 24, 26, 27)	Wrong motives (17) Self‐efficacy (11)
Attributions	External attribution (11)	Internal (and stable) attribution (14, 19)
Attitudes	Optimistic attitude (8) Friendly understanding attitude (10)	Harsh‐dominant‐resentful attitude (10)
(Coping with) emotions	Positive emotions (11) Avoidance coping (11)	Negative emotions (11, 17, 19, 20, 26) Stress (20, 24) Emotion‐focused coping (11) Critical expressed emotion (9)
Context		
Group size	Size of client group (3, 17) Small‐scale services (1)	Large services (1)
Team	Vision (11, 20, 24) Focus on all aspects of clients’ life (20, 24) Open culture (20, 24) Stability (24) Use of video feedback in meetings (27)	Focus on controlling/managing challenging behaviour (20, 24) Conflicts (20, 24) Lack of continuity (19, 20, 24)
Organization	Focus on staff *and* client (24) Open culture (24) Support (18, 19, 24, 25)	Focus on client *or* staff (24) Conflicts (20) Instability (24)
Relatives	Staff‐relative interactions (23)	

Note

Numbers refer to article numbers in Tables 1 and 2.

#### 3.2.1 Client level

##### Behaviour

The general occurrence, severity and type of challenging behaviour influenced interactions. Staff interacted less showed more negative attention and less positive, neutral and assisting attention, more avoidance‐related and assertive behaviour, and less engagement‐related and friendly behaviour towards clients with challenging behaviour when compared to clients without challenging behaviour (Emerson et al., 1992; Felce & Perry, 1995; Willems et al., 2010, 2014; Zijlmans, Embregts, Gerits, Bosman, & Derksen, 2014a, 2014b). The rate of severe challenging behaviour was positively associated with the rate of staff contact outside staff contact aimed at managing challenging behaviour (Emerson et al., 1992). The severity of specifically self‐injurious behaviour negatively influenced the quality of staffs’ interaction with clients outside the direct occurrence of self‐injurious behaviour. That is, the increased staff contact towards people with more severe behaviour was more likely to be in the form of a demand (Wolff et al., 2012). Zijlmans et al. (2012) showed a differential effect of internalizing and externalizing challenging behaviour (i.e. more controlling and hostile behaviour towards externalizing challenging behaviour compared to internalizing or both types of challenging behaviour). On the other hand, Willems et al. (2016) showed that only internalizing challenging behaviour had a significant influence on staff interactive behaviour (i.e. more friendly behaviour, less controlling behaviour).

The social behaviour of clients facilitated interactions. Clients’ warm and friendly behaviour was positively associated with friendly staff behaviour (Willems et al., 2016), and staff was more likely to interact with clients who were engaged in social activities rather than in non‐social or were disengaged (Felce et al., 1995). Clients who were able to initialize contact (Zijlmans et al., 2014a, 2014b) and make themselves understood (e.g. sign language, own type of language) facilitated interactions (Antonsson et al., 2008).

##### Emotions

Clients’ feelings of safety, security and confidence, which were strengthened by daily routines, facilitated interactions (Antonsson et al., 2008, 2013; Bambara et al., 2001; Nagra et al., 2017). It was important for clients to be able to express their emotions to staff (Antonsson et al., 2013; Nagra et al., 2017) and receive support to regulate (distressing) emotions (Clarke et al., 2019).

##### (Dis)abilities

Staff interacted more often with more able clients—in terms of adaptive behaviour, social and physical (in)capacity, and speech, self‐help and literacy skills—(Felce & Perry, 1995). Clients with severe intellectual disabilities experienced the lowest levels of staff contact (Zijlmans et al., 2014a, 2014b). Nevertheless, staff reported less friendly and more hostile behaviour towards clients with less severe intellectual disabilities (Willems et al., 2016).

#### 3.2.2 Staff level

##### Interactive principles

Zijlmans, Embregts, Gerits, Bosman, and Derksen (2014b) and Embregts, Kroezen, et al. (2019) operationalized successful interactions as those that satisfy clients’ needs for autonomy, relatedness and competence (i.e. self‐determination theory: Deci & Ryan, 1985/2000). Video feedback supported staff to attune to the different needs of clients (Thompson et al., 2019). First, the need for autonomy refers to respecting clients’ opinions and wishes. The feeling of autonomy was enhanced when staff encouraged clients to take initiative or decisions, followed clients’ lead and minimized control and pressure (Antonsson et al., 2013; Bambara et al., 2001; Embregts, Zijlmans, et al., 2019; Whittington & Burns, 2005; Zijlmans et al., 2014b). Nonetheless, clients’ wishes should be fulfilled within reasonable limits, and good relationships entailed having clear, explicit boundaries (Antonsson et al., 2008, 2013; Bambara et al., 2001; Bradshaw & Goldbart, 2013; Olivier‐Pijpers et al., 2020; Whittington & Burns, 2005). Second, the need for relatedness requires adequate responses to emotional signals and needs. Clients described feeling understood by staff as an important aspect of relationships with staff (Clarke et al., 2019). A failure to understand cues and respond adequately to clients’ emotional needs hinders interactions (Antonsson et al., 2008; Bambara et al., 2001; Embregts, Zijlmans, et al., 2019; Nagra et al., 2017; Zijlmans et al., 2014b). Personal ways to communicate enhanced feelings of relatedness (e.g. scratching the client's back, imitating one another: Antonsson et al., 2008) and staff should take time to truly understand a client, beyond his or her challenging behaviour (Olivier‐Pijpers et al., 2020). Third, the need for competence refers to supporting clients to perform activities on their own. Bradshaw and Goldbart (2013) showed that doing things for a client that the client can do himself hindered interactions. The feeling of competence was enhanced when staff gave clients the time and space to do tasks their way and to express themselves (Antonsson et al., 2008, 2013; Embregts, Zijlmans, et al., 2019; Nagra et al., 2017; Zijlmans et al., 2014b). McLaughlin and Carr (2005) showed that a staff training aimed at building rapport ameliorates relationships between staff and clients by increasing responsivity (as well as reciprocity, see interactive principle *reciprocity*). Responsivity was built by encouraging staff to acknowledge all communication attempt, to assess the function of the communication and to address the identified needs/requests when possible.

Another interactive principle included confirmation. Interactions thrived when staff confirmed clients’ actions (e.g. showing a genuine interest) and paid attention to all forms of communicative behaviour (Antonsson et al., 2013; Bambara et al., 2001). Confirming clients’ humanity was also an essential principle, referring to seeing the client as a person with characteristics and needs similar to themselves as well as recognizing clients’ individuality (Antonsson et al., 2013; Bambara et al., 2001; Bradshaw & Goldbart, 2013; Knotter et al., 2018).

Humour enhanced creating mutuality, contact and a positive interaction (Antonsson et al., 2008; Nagra et al., 2017).

In the study of Bambara et al. (2001), staff reflected on what it means to be trustworthy when building meaningful interactions with clients. Clients should know that staff is there for them, and staff would not leave because of their challenging behaviour (Bambara et al., 2001; Ravoux et al., 2012). Clients emphasized the importance of trust as well, referring to the approachability of staff for advice, guidance and support (Clarke et al., 2019; Olivier‐Pijpers et al., 2020).

Successful interactions were characterized by proximity. Staff drew analogies to friendships and family relationships, and some staff members reported that clients became part of their personal lives (Antonsson et al., 2008; Bambara et al., 2001).

Lastly, synchronization and reciprocity affected interactions. Staff who acted less dominant or more friendly stimulated clients to react with less dominance and more friendliness (Willems et al., 2016; Willems et al., 2018. Additionally, positive two‐way interactions strengthened the bond between staff and clients. Staff encouraged clients to reciprocate interaction by reinforcing their interaction attempts and providing them with the time and space they need to communicate (Antonsson et al., 2013; Nagra et al., 2017). McLaughlin and Carr (2005) showed that a staff training aimed at building rapport ameliorates relationships between staff and clients by increasing reciprocity (as well as responsivity, see interactive principle *satisfying clients’ needs*). Reciprocity was increased by encouraging staff to perform mutual preferred activities and share equally in the steps necessary to perform the activities.

##### Knowledge

Several studies referred to knowledge as an essential factor in building staff–client interactions. Besides knowledge about the nature of a treatment programme (Knotter et al., 2018), knowledge mainly referred to gaining personal knowledge about a specific client and getting to know and understand the client by establishing personal relationships (Antonsson et al., 2008, 2013, 2016; Bambara et al., 2001; Bradshaw & Goldbart, 2013; Ravoux et al., 2012; Whittington & Burns, 2005). Personal relationships were facilitated by spending time together (Antonsson et al., 2013; Bambara et al., 2001; Bradshaw & Goldbart, 2013), knowing a clients’ life history and daily life experiences (Antonsson et al., 2008, 2013; Knotter et al., 2018), and to some degree by talking to or observing others interact with the client (Bradshaw & Goldbart, 2013; Ravoux et al., 2012; Whittington & Burns, 2005).

##### Psychological resources

The motivation to work with clients with intellectual disabilities and challenging behaviour affected staff–client interactions. Salary as the main motivator to work caused conflicts, while enthusiastic staff who truly cared about clients facilitated staff–client interactions (Antonsson et al., 2008; Knotter et al., 2018). The relationship with a client added to staffs’ motivation to keep working with a client (Bambara et al., 2001).

Patience and flexibility incited interactions, enabling staff to give clients the necessary time and space (Antonsson et al., 2008, 2013; Bambara et al., 2001; Knotter et al., 2018; Nagra et al., 2017).

Confidence appeared to be another important factor in meaningful interactions (Antonsson et al., 2016; Nagra et al., 2017; Ravoux et al., 2012; Thompson et al., 2019). Willems et al. (2016) showed the positive effect of self‐efficacy (i.e. feeling competent and knowing your strengths) on friendly behaviour while some positive effect on control and hostility was reported as well.

Self‐reflection refers to staffs’ ability to reflect on the quality of care and the impact of their behaviour on clients. Self‐reflection helped staff learn from experiences with a client (Knotter et al., 2018; Ravoux et al., 2012), which was related to less hostile and controlling behaviour (Willems et al., 2016). Video feedback increased staffs’ ability to self‐reflect (Thompson et al., 2019).

##### Attributions

The causal explanations staff used to explain challenging behaviour (i.e. attributions) were discussed in relation to staff interactive behaviour. Two dimensions of attributions include controllability—the belief that a client can regulate his challenging behaviour (internal control) or that others can regulate challenging behaviour (external control), and stability—the belief that challenging behaviour is invariant or changeable. Zijlmans et al. (2012) showed that staff who utilized attributions of both internal control and stability showed more hostile, support‐seeking and controlling behaviour. Attributions were unrelated to friendly behaviour. In the study of Antonsson et al. (2016), carers reported that a decrease in internal attributions of control improved their interaction with clients. At the same time, Willems et al. (2016) did not find an effect of internal controllability on hostile behaviour, but did find a positive effect of external controllability on friendly behaviour.

##### Attitudes

Staffs’ optimistic and friendly understanding attitude towards clients facilitated interactions and friendly staff behaviour (Walz & Goldstein, 1992; Willems et al., 2014), while a harsh‐dominant‐resentful attitude predicted hostile staff behaviour, but also lower controlling behaviour (Willems et al., 2014).

##### (Coping with) emotions

Several studies included emotions as a predictor of staff–client interactions and staff interactive behaviour, including positive (e.g. cheerful, confident, excited or relaxed) and negative (e.g. anger, anxiety, depression, fear, irritation or uncertainty) emotions. Staff reported that negative emotions and stress resulting from clients’ challenging behaviour would normally hinder successful interactions (Antonsson et al., 2008, 2016; Bambara et al., 2001; Knotter et al., 2018; Ravoux et al., 2012; Whittington & Burns, 2005) and expressed a need for support to manage negative emotions (Antonsson et al., 2016; Whittington & Burns, 2005). However, Zijlmans et al. (2012) showed no added effect of negative emotions after adding types of challenging behaviour and attributions to the equation for controlling and hostile staff behaviour, and no relationship between negative emotions and friendly behaviour. Willems et al. (2016) showed that—apart from challenging behaviour and attributions—negative emotions were related to more controlling and hostile staff behaviour and positive emotions were associated with friendly behaviour.

Regulating emotions by looking for a distraction or the company of others (i.e. avoidance‐focused coping) rather than using anxious, angry and fantasy strategies (i.e. emotion‐focused coping) was associated with friendly staff behaviour (Willems et al., 2016). A tendency of staff to engage in negative emotional behaviour towards clients (e.g. criticism, unreasonable, cynical) increased hostility and diminished friendliness towards clients (Willems et al., 2010).

Staffs’ negative feelings became less prominent over time as sympathy increased by getting to know the client, indicating a habituation process (Ravoux et al., 2012; Whittington & Burns, 2005).

#### 3.2.3 Context level

##### Group size

The rate of staff contact was higher in smaller‐scale, more specialized settings when compared to larger services (Allen & Hill‐Tout, 1999), and interaction was facilitated in community settings that were genuinely small and based on ordinary housing (Felce et al., 1995). The size of a client group impacted interactions, rather than staff–client ratios. Staff spent the greatest proportion of time interacting with clients in a small group with the staff–client ratio of one to four (Felce et al., 1991). Higher staff–client ratios did not increase staff–client interactions in small or large groups (Felce et al., 1991, 1995; Felce & Perry, 1995). Staff experienced that it was easier to handle challenging behaviour when there were fewer carers on duty (Antonsson et al., 2008).

##### Team

First, cooperation and communication within teams appeared to be necessary for establishing meaningful staff–client interactions. A clear and coherent team vision promoted interactions and friendly behaviour, which included unified beliefs and a shared understanding of clients’ needs, reasons for challenging behaviour and support strategies. It was important for team members to share observations and insights about clients, and reflect on their actions (Bambara et al., 2001; Knotter et al., 2018; Willems et al., 2016). Teams focused on managing challenging behaviour, rather than on clients’ needs and all aspects of clients’ lives, reinforced negative interactions (Bambara et al., 2001; Knotter et al., 2018).

Second, an open team culture where everyone is free to express feelings, ideas and concerns facilitated staff–client interactions. Conflicts among team members impeded positive interactions, while support from colleagues facilitated interactions (Bambara et al., 2001; Knotter et al., 2018). Furthermore, an open culture included a team that shared responsibilities and accepted help from experts outside the team as well as from clients’ families. Outside professionals should share the team's beliefs and values; otherwise, interdisciplinary cooperation could hinder adequate support (Bambara et al., 2001; Knotter et al., 2018).

Third, staff turnover and temporary employers hindered interactions (Antonsson et al., 2016; Bambara et al., 2001; Knotter et al., 2018).

##### Organization

The organizational culture and vision affected staffs’ ability to engage in meaningful staff–client interactions. A strong organizational focus on either clients’ needs *or* staff safety hampered interactions, while attention for staff and client well‐being facilitated interactions (Knotter et al., 2018). Open communication within the organization, and support and recognition from management enabled interactions (Antonsson et al., 2013, 2016; Knotter et al., 2018; Nagra et al., 2017; Olivier‐Pijpers et al., 2020). Importantly, management actions and staff beliefs should correspond (Bambara et al., 2001). Frequent changing managers, behavioural experts or organizational visions obstructed interactions (Knotter et al., 2018).

##### Relatives

Besides meaningful interactions between staff and clients, between team members and within organizations, positive interactions between staff and clients’ relatives are essential to provide quality support. These interactions do contribute to meaningful staff–client interactions. However, contact between staff and relatives is not self‐evident (Olivier‐Pijpers et al., 2020).

## 4 DISCUSSION

**Figure 2:**
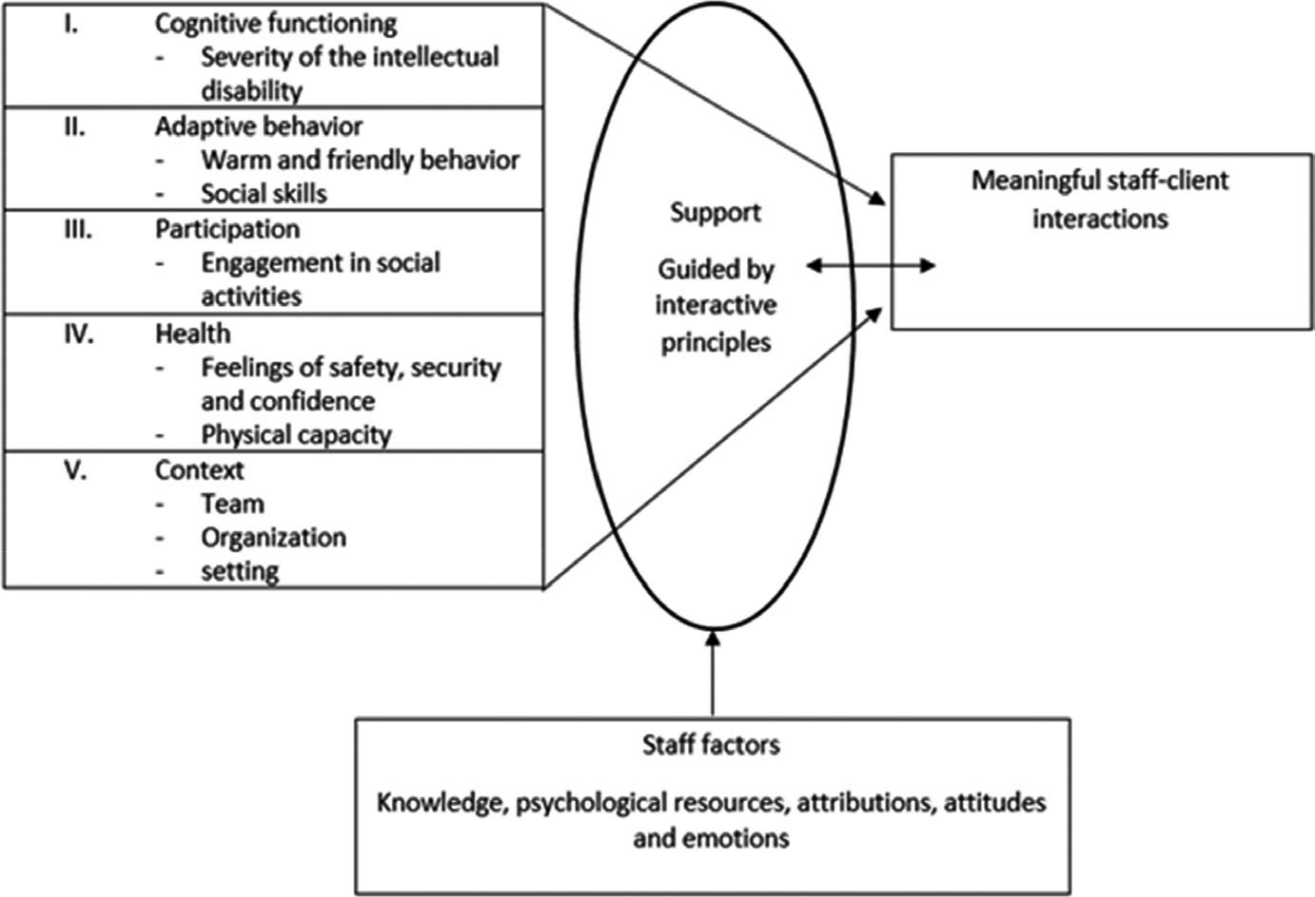
The factors identified in the present review presented within the adapted AAIDD‐model (Embregts, Kroezen, et al.,^2019^)

The present review provided an overview of the quantitative and qualitative literature regarding factors at the client, staff and context level that affect meaningful interactions between staff and clients with borderline to profound intellectual disabilities and challenging behaviour. The results are in line with the AAIDD‐model which suggests that the individual functioning of people with intellectual disabilities results from an interplay between personal and contextual factors combined with the support that clients receive (Embregts, Kroezen, et al., 2019). In Figure 2, the factors identified in the present review have been included in the adapted AAIDD‐model as published by Embregts, Zijlmans, et al. (2019) to provide a comprehensive overview of the results and their interconnectedness. Within the model, it is important that staff support fits clients’ needs within the five dimensions. The five dimensions have been operationalized with factors identified in the present study. The interactive principles may guide staff to provide this support in a manner that results in meaningful interactions. We have added staff factors to the model as these factors impact staffs’ ability to engage in meaningful interactions. Willems has presented a more elaborate model of the impact of staff factors on staff interactive behaviour and the importance of each factor (see Willems, 2016). The following discussion offers a brief overview of the identified factors per level and considers results in light of previous research.

At the client level, behaviour, emotions and (dis)abilities influenced staff–client interactions. The severity of clients’ intellectual disability was associated with increased quality and decreased quantity of staff–client interactions. According to Weiner's revised attribution model (1995), staff hold clients with less severe intellectual disabilities more responsible for challenging behaviour compared to clients with more severe intellectual disabilities and may approach clients with more severe intellectual disabilities with more sympathy, accounting for the positive influence of the severity of intellectual disabilities on the quality of staff–client interactions (Willems, 2016). Simultaneously, the severity of intellectual disabilities is typically associated with social skill deficits, decreasing frequencies of interactions (Duncan et al., 1999). The presence of challenging behaviour, compared to no challenging behaviour, lowered the frequency and quality of staff–client interactions as well, putting clients with more severe intellectual disabilities and challenging behaviour at high risk for experiencing low levels of staff contact.

At the staff level, interactive principles, knowledge, psychological resources, attributions, attitudes and (coping with) emotions influenced staff–client interactions. It is generally acknowledged that staff need training to offer adequate support to clients with intellectual disabilities and challenging behaviour, and many of the staff level factors have been shown trainable in previous research (e.g. attitudes, coping strategies, self‐reflection, confidence, communication, attributions). Yet, staff–client relationships are not always part of training programmes (Van Oorsouw et al., 2013).

One of the interactive principles concerned satisfying clients’ need for autonomy. While autonomy facilitated interactions, challenging behaviour puts clients at risk for experiencing little autonomy. Making choices can be stressful for clients, especially when choices do not fit clients’ needs (Stalker & Harris, 1998). Therefore, guidance and setting boundaries that fit clients’ needs are essential, and staff should find a balance between their autonomy‐giving and controlling interactive behaviour (Willems, 2016).

Previous research has suggested an association between clients’ challenging behaviour and staffs’ negative emotions (Hastings, 2005). In the present review, studies using qualitative methodologies reported that these negative emotions hindered interactions. The results of quantitative studies were somewhat inconsistent. While Zijlmans et al. (2012) reported no (added) effect of negative emotions on staff interactive behaviours, Willems et al. (2010), Willems et al. (2016) reported a clear negative effect of negative emotions and a positive effect of positive emotions on staff interactive behaviours in a later study with more participants including positive alongside negative emotions. The significance of emotions appeared much higher compared to attributions (Willems et al., 2016), suggesting that emotions have a more immediate effect compared to cognitive processes (Willems, 2016). The actual impact of negative emotions is moderated by coping strategies (Hastings, 2005). Avoidance‐focused coping facilitated interactions, underpinning the importance of open team and organization cultures in which colleagues support each other to cope with emotions.

Results were mixed for the effect of experience on staff–client interactions: negative (Walz & Goldstein, 1992), positive (Willems et al., 2010) and no effects (Willems et al., 2016) were reported. Mere experience seems insufficient to facilitate interactions as regular encounters between staff and clients do not necessarily produce the expertise required to understand a client. Rather, gaining professional knowledge depends on staffs’ interest in and intentionality of getting to know the client, and the process of getting to know and understand a client seems to count rather than mere years of experience (Reinders, 2010; Schuengel et al., 2010).

At the context level, we identified group size, team and organization factors. Building interpersonal relationships with colleagues seems just as important as building interpersonal relationships with clients. In previous research, organizational factors were more strongly associated with staffs’ well‐being compared to clients’ challenging behaviour or other characteristics (Hastings, 2005).

### 4.1 Quality of included studies

Quality of the included studies was assessed independently by two reviewers (LR, MS) using the checklist constructed by Schepens et al. (2018)^1^. Inter‐rater agreeability was 85%. Unfortunately, there was only limited variation between the studies in quality scores which did not allow us to use a meaningful weighting procedure to reflect more confidence in papers with higher quality scores or to exclude papers based on quality. Therefore, the quality assessment had no further implications for the results nor their interpretation and the assessment was excluded from the methods and results sections. Results from the quality assessment are available from the first author. More sensitive criteria are necessary to assess the quality of quantitative, qualitative and mixed‐method studies in future literature reviews.

### 4.2 Strengths, limitations and implications for future research

The inclusion of both quantitative and qualitative research strengthened the present review. Although there is some criticism on synthesizing qualitative literature, stemming from the belief that results are de‐contextualized and not generalizable, the inclusion of qualitative research ensures that findings are grounded in the experiences of involved parties.

Two recent studies published in 2019 and 2020 included in the present review considered insights from clients (Clarke et al., 2019; Olivier‐Pijpers et al., 2020). Dodevska and Vassos (2013) showed that professionals and clients conceptualize a ‘good’ staff member differently, underpinning the importance of persevering this emerging trend of including clients’ insights in future research into staff–client interactions.

From the included studies, it was impossible to categorize factors per levels of intellectual disabilities. As levels of intellectual disabilities were indicated to influence the quality and quantity of staff–client interactions, it is important to address staff–client interactions per client group in future research.

Diverse conceptualizations of ‘staff–client interactions’ appeared in the literature. Studies evaluating the successfulness of interaction applied various conceptualizations of ‘successful’ (e.g. encourage participation in activity or interaction, fulfil clients’ needs, manage challenging behaviour, affect quality of life). Other studies addressed interactive behaviour without referring to the desirability of behaviours. Therefore, the division between facilitating and hindering factors is arbitrary and not empirically tested.

From the literature base of the present review, it was impossible to study the dependency between variables while it is presumable that client, staff and contextual factors work together to influence staff–client interactions (AAIDD‐model, Embregts, Kroezen, et al., 2019). The interplay within and between the (levels of) factors identified in the present review would be interesting to explore in future research. In addition, as illustrated in Table 4, there is a widely varying amount of research on the different factors and evidence on some factors was contradictory. It is important to keep in mind the strength of evidence for each of the factors identified in the present review when considering the factors in future research and practice.

### 4.3 Implications for practice

As depicted in Figure 2, meaningful interactions result from a balance between the five dimensions combined with adequate support. To enhance meaningful interactions in practice, one identifies the support needs of a client in each dimension and fits the support to the needs within the dimensions. The interactive principles guide staff to shape the support in a way that promotes meaningful interactions. The staff factors are important starting points to check whether staff are sufficiently equipped to engage in meaningful interactions.

## CONFLICT OF INTEREST

The authors have no competing interests to declare.

## AUTHOR CONTRIBUTIONS

All authors have contributed to, seen, and approved of the manuscript and agree to the order of authors as listed on the title page.

## Footnotes

^1^ The checklist is available from the first author, H.R.M.M. Schepens.

## References

[jar12830-bib-0001] Allen, D. (2001). Mediator analysis: An overview of recent research on carers supporting people with intellectual disability and challenging behaviour. Journal of Intellectual Disability Research, 43(4), 325–339. 10.1046/j.1365-2788.1999.00209.x 10466871

[jar12830-bib-0002] Allen, D. , & Hill‐Tout, J. (1999). A day in the life: Day activities for people with intellectual disabilities and challenging behaviour in two English counties. Journal of Applied Research in Intellectual Disabilities, 12(1), 30–45. 10.1111/j.1468-3148.1999.tb00048.x

[jar12830-bib-0003] Antonsson, H. , Åström, S. , Lundström, M. , & Grandeheim, U. H. (2013). Skilled interaction among professional carers in special accommodations for adult people with learning disabilities. Journal of Psychiatric and Mental Health Nursing, 20(7), 576–583. 10.1111/j.1365-2850.2012.01934.x 22676335

[jar12830-bib-0004] Antonsson, H. , Graneheim, U. H. , Isaksson, U. , Åström, S. , & Lundström, M. O. (2016). Evaluation of a web‐based training program for professional carers working with people with learning disabilities and challenging behaviour: A pilot study with SSED‐Design. Issues in Mental Health Nursing, 37(10), 734–743. 10.1080/01612840.2016.1189636 27351080

[jar12830-bib-0005] Antonsson, H. , Graneheim, U. H. , Lundström, M. , & Strm, S. (2008). Caregivers’ reflections on their interactions with adult people with learning disabilities. Journal of Psychiatric and Mental Health Nursing, 15(6), 484–491. 10.1111/j.1365-2850.2008.01259.x 18638209

[jar12830-bib-0006] Bambara, L. M. , Gomez, O. , Koger, F. , Lohrmann‐O’Rourke, S. , & Xin, Y. P. (2001). More than techniques: Team members’ perspectives on implementing positive supports for adults with severe challenging behaviors. Journal of the Association for Persons with Severe Handicaps, 26(4), 213–228. 10.2511/rpsd.26.4.213

[jar12830-bib-0007] Bradshaw, J. , & Goldbart, J. (2013). Staff views of the importance of relationships for knowledge development: Is training by specialists a waste of money? Journal of Applied Research in Intellectual Disabilities, 26(4), 284–298. 10.1111/jar.12020 23386258

[jar12830-bib-0008] Burton, M. E. H. (2001). Understanding and responding to behavioural challenges: An investigative approach. Manchester Learning Disability Partnership.

[jar12830-bib-0009] Carr, A. , Linehan, C. , O'Reilly, G. , Walsh, P. N. , & McEvoy, J. (2016). The handbook of intellectual disability and clinical psychology practice (2 London: nd ed.). Taylor & Francis Ltd.

[jar12830-bib-0010] Clarke, A. , Dagnan, D. , & Smith, I. C. (2019). How service‐users with intellectual disabilities understand challenging behaviour and approaches to managing it. Journal of Applied Research in Intellectual Disabilities, 32(5), 1–13. 10.1111/jar.12612 31066173

[jar12830-bib-0011] Cox, A. D. , Dube, C. , & Temple, B. (2015). The influence of staff training on challenging behaviour in individuals with intellectual disability: A review. Journal of Intellectual Disabilities, 19(1), 69–82. 10.1177/1744629514558075 25395332

[jar12830-bib-0012] Deci, E. L. , & Ryan, R. M. (2000). The “what” and “why” of goal pursuits: Human needs and the self‐determination of behaviour. Psychological Inquiry, 11(4), 227–268. 10.1207/S15327965PLI1104_01

[jar12830-bib-0013] Dodevska, G. A. , & Vassos, M. V. (2013). What qualities are valued in residential direct care workers from the perspective of people with an intellectual disability and managers of accommodation services? Journal of Intellectual Disability Research, 57(7), 601–615. 10.1111/j.1365-2788.2012.01565.x 22563721

[jar12830-bib-0014] Duncan, D. , Matson, J. L. , Bamburg, J. W. , Cherry, K. E. , & Buckley, T. (1999). The relationship of self‐injurious behavior and aggression to social skills in persons with severe and profound learning disability. Research in Developmental Disabilities, 20(6), 441–448. 10.1016/S0891-4222(99)00024-4 10641253

[jar12830-bib-0015] Embregts, P. , Kroezen, M. , Mulder, E. J. , Van Bussel, C. , Van der Nagel, J. , Budding, M. , Wieland, J. (2019). *Multidisciplinaire richtlijn probleemgedrag bij volwassenen met een verstandelijke beperking* . [Multidisciplinary guideline challenging behaviour in adults with an intellectual disability]. NVAVG.

[jar12830-bib-0016] Embregts, P. J. C. M. , Zijlmans, L. J. M. , Gerits, L. , & Bosman, A. M. T. (2019). Evaluating a staff training program on the interaction between staff and people with intellectual disability and challenging behaviour: An observational study. Journal of Intellectual & Developmental Disability, 44(2), 131–138. 10.3109/13668250.2017.1350839

[jar12830-bib-0017] Emerson, E. , Beasley, F. , Offord, G. , & Mansell, J. (1992). An evaluation of hospital‐based specialized staffed housing for people with seriously challenging behaviours. Journal of Intellectual Disability Research, 36(Pt 4), 291–307. 10.1111/j.1365-2788.1992.tb00529.x 1525438

[jar12830-bib-0018] Felce, D. , Lowe, K. , & Blackman, D. (1995). Resident behaviour and staff interaction with people with intellectual disabilities and seriously challenging behaviour in residential services. Mental Handicap Research, 8(4), 272–295. 10.1111/j.1468-3148.1995.tb00162.x

[jar12830-bib-0019] Felce, D. , & Perry, J. (1995). The extent of support for ordinary living provided in staffed housing: the relationship between staffing levels, resident characteristics, staff:resident interactions and resident activity patterns. Social Science & Medicine, 40(6), 799–810. 10.1016/0277-9536(94)00152-J 7747214

[jar12830-bib-0020] Felce, D. , Repp, A. C. , Thomas, M. , Ager, A. , & Blunden, R. (1991). The relationship of staff:client ratios, interactions, and residential placement. Research in Developmental Disabilities, 12(3), 315–331. 10.1016/0891-4222(91)90015-K 1792360

[jar12830-bib-0021] Hastings, R. P. (2005). Staff in special education settings and behaviour problems: Towards a framework for research and practice. Educational Psychology, 25(2–3), 207–221. 10.1080/0144341042000301166

[jar12830-bib-0022] Hastings, R. P. , Allen, D. , Baker, P. A. , Gore, N. J. , Hughes, J. C. , McGill, P. , & Toogood, S. (2013). A conceptual framework for understanding why challenging behaviours occur in people with developmental disabilities. International Journal of Positive Behavioural Support, 3(2).5–12.

[jar12830-bib-0023] Hastings, R. P. , & Remington, B. (1994). Staff behaviour and its implications for people with learning disabilities and challenging behaviours. British Journal of Clinical Psychology, 33(4), 423–438. 10.1111/j.2044-8260.1994.tb01140.x 7874036

[jar12830-bib-0024] Healthcare Inspectorate . (2005). Complexe gedragsproblematiek bij mensen met een ernstige verstandelijke handicap vereist bundeling van specialistische expertise. [Complex behavioural problems in people with severe intellectual disability require bundling specialist expertise]. .

[jar12830-bib-0025] Hermsen, M. A. , Embregst, P. J. , Hendriks, A. H. , & Frielink, N. (2014). The human degree of care. Professional loving care for people with a mild intellectual disability: An explorative study. Journal of Intellectual Disability Research, 58(3), 221–232. 10.1111/j.1365-2788.2012.01638.x 23057560

[jar12830-bib-0026] Kevan, F. (2003). Challenging behaviour and communication difficulties. British Journal of Learning Disabilities, 31(2), 75–80. 10.1046/j.1468-3156.2003.00226.x

[jar12830-bib-0027] Knotter, M. H. , Moonen, X. M. H. , Wissink, I. B. , Finkenflügel, H. J. M. , & Stams, G. J. J. M. (2018, in press).Antecedents of interactions between staff members and aggressive clients with ID: A qualitative study In M. H. Kotter (2019). The whole is more: A contextual perspective on attitudes and reactions of staff towards aggressive behaviour of clients with ID in residential institutions (Doctoral dissertation). University of Amsterdam.

[jar12830-bib-0028] McConkey, R. , Morris, I. , & Purcell, M. (1999). Communications between staff and adults with intellectual disabilities in naturally occurring settings. Journal of Intellectual Disability Research, 43(3), 194–205. 10.1046/j.1365-2788.1999.00191.x 10392606

[jar12830-bib-0029] McLaughlin, M. , & Carr, E. G. (2005). Quality of rapport as a setting event for problem behavior. Journal of Positive Behaviour Interventions, 7(2), 68–91. 10.1177/10983007050070020401

[jar12830-bib-0030] Nagra, M. K. , White, R. , Appiah, A. , & Rayner, K. (2017). Intensive interaction training for paid carers: ‘Looking, looking and find out when they want to relate to you’. Journal of Applied Research in Intellectual Disability, 30(4), 648–660. 10.1111/jar.12259 27279387

[jar12830-bib-0031] Olivier‐Pijpers, V. C. , Cramm, J. M. , & Nieboer, A. P. (2020). Residents’ and resident representatives’ perspectives on the influence of the organisational environment on challenging behaviour. Research in Developmental Disabilities, 100 10.1016/j.ridd.2020.103629 32142969

[jar12830-bib-0032] Ravoux, P. , Baker, P. , & Brown, H. (2012). Thinking on your feet: Understanding the immediate responses of staff to adults who challenge intellectual disability services. Journal of Applied Research in Intellectual Disabilities, 25(3), 189–202. 10.1111/j.1468-3148.2011.00653.x 22489031

[jar12830-bib-0033] Reinders, H. (2010). The importance of tacit knowledge in practices of care. Journal of Intellectual Disability Research, 54(S1), 28–37. 10.1111/j.1365-2788.2009.01235.x 20586882

[jar12830-bib-0035] Schalock, R. L. (2004). The concept of quality of life: What we know and do not know. Journal of Intellectual Disability Research, 48(Pt3), 203–216. 10.1111/j.1365-2788.2003.00558.x 15025663

[jar12830-bib-0036] Schalock, R. L. , Borthwick‐Duffy, S. A. , Bradley, V. J. , Buntinx, W. H. E. , Coulter, D. L. , Craig, E. P. , & Yeager, M. H. (2010). Intellectual disability: Definition, classification, and systems of support (11th ed.). American Association on Intellectual & Developmental Disabilities.

[jar12830-bib-0037] Schepens, R. M. M. , Van Puyenbroeck, J. , & Maes, B. (2018). How to improve the quality of life of elderly people with intellectual disability: A systematic literature review of support strategies. Journal of Applied Research in Intellectual Disabilities, 32(3), 483–521. 10.1111/jar.12559 30575226

[jar12830-bib-0038] Schuengel, C. , Kef, S. , Damen, S. , & Worm, M. (2010). ‘People who need people’: Attachment and professional caregiving. Journal of Intellectual Disability Research, 54(Suppl 1), 38–47. 10.1111/j.1365-2788.2009.01236.x 20586883

[jar12830-bib-0039] Stalker, K. , & Harris, P. (1998). The exercise of choice by adults with intellectual disabilities: A literature review. Journal of Applied Research in Intellectual Disabilities, 11(1), 60–76. 10.1111/j.1468-3148.1998.tb00034.x

[jar12830-bib-0040] Thomas, J. , & Harden, A. (2008). Methods for the thematic synthesis of qualitative research in systematic reviews. BMC Medical Research Methodology, 8(1), 45–55. 10.1186/1471-2288-8-45 18616818 PMC2478656

[jar12830-bib-0041] Thompson, B. , Tickle, A. , & Dillon, G. (2019). Discovery awareness for staff supporting individuals with intellectual disabilities and challenging behaviour: Is it helpful and does it increase self‐efficacy? International Journal of Developmental Disabilities, 1–14. 10.1080/20473869.2019.1599605 PMC7942778 34141398

[jar12830-bib-0042] Van Oorsouw, W. M. W. J. , Embregts, P. J. C. M. , & Bosman, A. M. T. (2013). Evaluating staff training: Taking account of interactions between staff and clients with intellectual disability and challenging behaviour. Journal of Intellectual & Developmental Disability, 38(4), 356–364. 24279788 10.3109/13668250.2013.826787

[jar12830-bib-0043] Walz, L. , & Goldstein, L. H. (1992). The mental impairment and evaluation treatment service: Staff attitudes and staff‐client interactions. Psychological Medicine, 22(2), 503–511. 10.1017/S0033291700030440 1615116

[jar12830-bib-0044] Weiner, B. (1995). Judgments of responsibility: A foundation for a theory of social conduct. The Guilford Press.

[jar12830-bib-0045] Whittington, A. , & Burns, J. (2005). The dilemmas of residential care staff working with the challenging behaviour of people with learning disabilities. British Journal of Clinical Psychology, 44(Pt 1), 59–76. 10.1348/014466504X19415 15826344

[jar12830-bib-0046] Willems, A. P. A. M. (2016). Challenging relationships: Staff interactions in supporting persons with intellectual disabilities and challenging behaviour (Doctoral Dissertation). Tilburg University, Datawise/University Press Maastricht.

[jar12830-bib-0047] Willems, A. P. , Embregts, P. J. , Bosman, A. M. , & Hendriks, A. H. (2014). The analysis of challenging relations: Influences on interactive behaviour of staff towards clients with intellectual disabilities. Journal of Intellectual Disability Research, 58(11), 1072–1082. 10.1111/jir.12027 23480642

[jar12830-bib-0048] Willems, A. , Embregts, P. , Hendriks, L. , & Bosman, A. (2016). Towards a framework in interaction training for staff working with clients with intellectual disabilities and challenging behaviour. Journal of Intellectual Disability Research, 60(2), 134–148. 10.1111/jir.12249 26708920

[jar12830-bib-0049] Willems, A. P. A. M. , Embregts, P. J. C. M. , Stams, G. J. J. M. , & Moonen, X. M. H. (2010). The relation between intrapersonal and interpersonal staff behaviour towards clients with ID and challenging behaviour: A validation study of the Staff‐Client Interactive Behaviour Inventory. Journal of Intellectual Disability Research, 54(1), 40–51. 10.1111/j.1365-2788.2009.01226.x 19912463

[jar12830-bib-0050] Willems, A. , Embregts, P. , Wijnants, M. , Hendriks, L. , & Bosman, A. (2018). Dynamic patterns of three staff members interacting with a client with an intellectual disability and challenging behaviour: Suggestions for coaching. Nonlinear Dynamics, Psychology, and Life Sciences, 22(4), 535–562. 30336798

[jar12830-bib-0051] Wolff, J. J. , Clary, J. , Harper, V. N. , Bodfish, J. W. , & Symons, F. J. (2012). Evidence for reciprocal interaction effects among adults with self‐injury and their caregivers. American Journal on Intellectual and Developmental Disabilities, 117(3), 225–232. 10.1352/1944-7558-117.3.225 22716264 PMC3709855

[jar12830-bib-0052] Zijlmans, L. J. M. , Embregts, P. J. C. M. , Bosman, A. M. T. , & Willems, A. P. A. M. (2012). The relationship among attributions, emotions, and interpersonal styles of staff working with clients with intellectual disabilities and challenging behavior. Research in Developmental Disabilities, 33(5), 1484–1494. 10.1016/j.ridd.2012.03.022 22522206

[jar12830-bib-0053] Zijlmans, L. , Embregts, P. , Gerits, L. , Bosman, A. , & Derksen, J. (2014a). Engagement and avoidance in support staff working with people with intellectual disability and challenging behaviour: A multiple‐case study. Journal of Intellectual & Developmental Disability, 39(3), 233–242. 10.3109/13668250.2014.918592

[jar12830-bib-0054] Zijlmans, L. J. M. , Embregts, P. J. C. M. , Gerits, L. , Bosman, A. M. T. , & Derksen, J. J. L. (2014b, unpublished). The effectiveness of staff training on the interaction between staff and clients with intellectual disabilities and challenging behaviour: an observational study In L. J. M. Zijlmans (2014). Knowing me, knowing you: On staff supporting people with intellectual disabilities and challenging behaviour (Doctoral Dissertation). Tilburg University. Ridderkerk: Ridderprint.

